# Multivariate Analysis as a Method to Evaluate Antigenic Relationships between Bovine Viral Diarrhea Virus 1b Isolates and Vaccine Strains

**DOI:** 10.3390/v15102085

**Published:** 2023-10-13

**Authors:** Shollie M. Falkenberg, Hao Ma, Eduardo Casas, Rohana P. Dassanayake, Michael W. Bolton, Gage Raithel, Scott Silvis, John D. Neill, Paul H. Walz

**Affiliations:** 1 Sugg Laboratory, Department of Pathobiology, College of Veterinary Medicine, Auburn University, Auburn, AL 36849, USAsds0047@auburn.edu (S.S.); walzpau@auburn.edu (P.H.W.); 2 Ruminant Disease and Immunology Research Unit, National Animal Disease Center, USDA, Agricultural Research Service, Ames, IA 50010, USAjdneill19@gmail.com (J.D.N.); 3 Spring Hill Farm Consulting, Honor, MI 49640, USA; boltonhops@gmail.com

**Keywords:** antigenicity, bovine viral diarrhea virus, genetic diversity, principal component analysis, virus-neutralizing titer

## Abstract

The antigenicity of bovine viral diarrhea virus (BVDV) has been evaluated using virus-neutralizing titer data analyzed by principal component analysis (PCA) and has demonstrated numerous isolates to be antigenically divergent from US vaccine strains. The lack of BVDV-1b strains in currently licensed vaccines has raised concerns regarding the lack of protection against BVDV-1b field strains. The aim of this study was to evaluate the antigenic diversity of BVDV-1b strains and better understand the breadth of antigenic relatedness using BVDV-1b antisera and antisera from vaccine strains. Results from this analysis demonstrate the antigenic diversity observed among BVDV-1b isolates and genetic assignment into the BVDV-1b subgenotype is not representative of antigenic relatedness. This is demonstrated by BVDV-1b isolates (2280N, SNc, Illc, MSU, and 2337) observed to be as antigenically dissimilar as BVDV-2a isolates when using BVDV-1b antisera. Additionally, when BVDV-1a vaccine antisera was used for comparisons, a greater percentage of BVDV-1b isolates clustered with BVDV-1a vaccine strains as part of PC1, suggesting antigenic relatedness and potentially partial protection. Collectively, data from this study would suggest that while most BVDV-1b isolates are antigenically similar, there are antigenically dissimilar BVDV-1b isolates as determined by the lack of cross-reactivity, which may contribute to the lack of protection.

## 1. Introduction

The bovine viral diarrhea virus (BVDV), belonging to the genus *Pestivirus* within the family *Flavivirdae*, is one of the most impactful viruses affecting the cattle industry. Phylogenetic analyses indicate that BVDV has been circulating in cattle populations for hundreds of years [1,2]. Currently, phylogenetic analysis has identified 22 BVDV-1 subgenotypes (BVDV-1a-u) and 4 BVDV-2 subgenotypes (BVDV-2a-d) globally, as classically described [3]. While there seems to be a growing genetic diversity of BVDV globally, the most prevalent subgenotypes worldwide are 1a, 1b, and 2a [3]. Currently, BVDV-1b strains predominate in the United States [4,5], and the initial predominance of BVDV-1b strains was first described in prevalence studies in the late 1990s [6]. Although previous prevalence studies in the late 1980s described BVDV-1a as the most prevalent BVDV subgenotype [6].

Emergence or a change in the prevalence of BVDV subgenotypes is clinically and biologically important, as providing protection against diverse BVDV strains poses potential issues due to antigenic diversity observed within and among BVDV species and subgenotypes.

Current BVDV vaccines contain strains from two (BVDV-1a and 2a) species. Given the prevalence of 1b strains, concern exists that current vaccines might not confer protection against different subtypes and antigenically divergent strains. This concern has been fueled by not only the increasing prevalence of BVDV-1b but also in vitro studies, which have demonstrated differences in monoclonal antibody binding between BVDV species [7,8], lower antibody titers against different *Pestivirus* species [9,10,11,12], lower antibody titers against different subgenotypes [8,13,14], and differences in antibody titers within subgenotypes [15]. These comparisons only accounted for the humoral component of the immune response and might not completely extrapolate into accurately characterizing efficacy.

Although differences in cell-mediated responses within subgenotypes have also been reported [16], it should be noted that each of these measures is an individual measurement and does not account for a holistic and coordinated immune response, similar to measures of just humoral immunity. This is highlighted by discordant data from experimental challenge studies, which suggest that currently licensed US vaccines have demonstrated protection against BVDV-1b challenge and exposure. Furthermore, data from antigenic comparison studies have suggested that there were no specific patterns associated with isolates belonging to the same subgenotype, but rather it was isolate dependent with respect to antigenically divergent strains [17,18,19].

Collectively, this highlights the need to better characterize antigenic differences among specifically BVDV-1b isolates in an effort to understand their increasing prevalence and the perceived lack of vaccination protection being observed in the field, as indicated by the predominance of BVDV-1b detection in diagnostic specimens. Therefore, the current study aimed to examine genetic and antigenic relationships among a total of 30 BVDV strains/isolates comprised of 11 noncytopathic BVDV-1b isolates that were isolated from dairy herds where persistently infected (PI) calves were born in herds utilizing typical vaccination protocols, 13 previously isolated BVDV-1b strains, three cytopathic BVDV-1a vaccine strains (C24V, NADL, and Singer), and three cytopathic BVDV-2a vaccine strains (125c, 296c, and 53637).

## 2. Materials and Methods

Procedures for the generation of BVDV-specific antisera followed previously published methodology [17,18,19], and procedures were reviewed and approved by the Institutional Animal Care and Use Committees at the National Animal Disease Center and Auburn University (protocols #ARS-2017–673 and #2018-3350, respectively). Calves were determined to be *Pestivirus* antibody and antigen negative prior to enrollment for antisera generation. All calves were maintained within their respective virus exposure group in BSL 2 isolation during the study period. Briefly, BVDV-naive calves were intranasally inoculated (2.5 mL/nostril) with approximately10^6^ CCID_50_ of each respective BVDV-1b isolate or vaccine strain. The calves were administered the same respective isolate or strain subcutaneously (5 mL) on study day 28, and antisera was harvested on day 56. Approximately twenty-four hours after blood collection, the serum was harvested and aliquoted for preservation.

A total of 30 viruses were propagated for this study and used for antisera generation and virus neutralization assay (Table 1). Cell maintenance and virus propagation are previously described [17,18,19] for 19 of the viruses used in the study that included the six vaccine strains (BVDV-1a strains C24V, NADL, Singer and BVDV-2a strains 125c, 296c, 53637) and 13 BVDV-1b viruses from the USDA-ARS-NADC viral inventory. Cell maintenance, virus isolation and propagation are previously described [20] for the 11 contemporary field BVDV-1b viruses isolated from persistently infected (PI) calves. Whole-blood and spleen samples were used for virus isolation from calves previously determined to be PI. Additionally, the 11 contemporary field BVDV PI viral isolates, designated by DV and MRD were obtained concurrently from two farms, demonstrating circulating diversity of BVDV-1b viruses within and among two herds.

Details regarding complete genome sequencing, detailed phylogeny, and BVDV isolate characterization are previously described in literature [17,18,19]. Briefly, in order to determine the amino acid differences within the E2 protein for the viruses used for generation of the phylogenetic tree, Clustal Omega alignment was performed and the maximum-likelihood method with the appropriate substitution model from Geneious Prime was used to obtain the phylogenetic tree with branch support estimated using 1000 bootstrap replicates. Phylogenic analysis was conducted using 80 BVDV isolates. This included the 11 contemporary field BVDV-1b viruses isolated from PI calves used for antisera generation, 13 BVDV-1b viruses from the viral collection at USDA-ARS-NADC used for antisera generation, 50 sequences obtained from GenBank submissions or sequences from the viral collection at USDA (Table S1), three cytopathic BVDV-1a, and three BVDV-2a vaccine strains that are currently contained in licensed BVDV vaccines for genetic comparisons and diversity.

Virus neutralization titer (VNT) assays were performed as previously outlined with minor modification [17,18,19], using the 30 viruses tested against the same 30 antisera in a 30 × 30 checkerboard testing method. Briefly, in cell culture 96-wells microplates, using replicates of five wells for each serum dilution, a 50 μL aliquot of diluted serum and a 50 μL aliquot of virus containing 250 TCID50 were added to each well and incubated for 1 h at 37 °C. At the end of the incubation period, approximately 20,000 MDBK cells (in a 100 μL aliquot of MEM and 10% equine serum) were added to each well. Microplates were incubated for 4 days at 37 °C in a 5% CO_2_ incubator. The noncytopathic viruses were stained using a combination of the monoclonal antibodies D89 (VMRD #D89) and 20.10.6 [21] at concentrations of 1 µg/mL and 5 µg/mL, respectively. A rabbit anti-mouse HRP conjugate (Jackson ImmunoResearch, West Grove, PA, USA; #315-035-003) was used to facilitate staining with AEC reagent (Enzo Life Sciences, Farmingdale, NY, USA; #ENZ-43825). For cytopathic (cp) isolates, wells without any observed cp effect or for non-cytopathic isolates the lack of cell layer staining in each serum dilution were used for the calculation of the endpoint through the Spearman–Kärber method, as previously described [8]. To ensure continuity among VN assays, viruses used for VN testing were propagated from the same passage titered and aliquoted for each use. Additionally, back titer plates were included for each VN assay to confirm TCID50.

The determined VNT were transformed into log2 values and used to generate distribution of the data represented by box and whisker plots and also to demonstrate the distribution of VNT for each respective analysis. VNT for each respective antiserum against each of the respective strains or isolate were used to determine average VNT for the respective antiserum. Principle component analysis (PCA) utilized for this study was conducted as previously described [17,18,19]. Three analyses were conducted using three different antisera groups which included all BVDV-1b antisera against all 30 viruses, BVDV-1a vaccine antisera against all 30 viruses, and BVDV-2a vaccine antisera against all 30 viruses.

PCA used in this study followed described methods [17,18,19], yielding two different illustrations of the data, both a PCA cluster dendrogram and scatter plot. The PCA dendrogram combined the variation from both the first PC (PC1) and second PC (PC2) into one value to cluster the strains and isolates into antigenically similar groups as determined by VNT. A height of 1 within the PCA cluster dendrogram was used as the minimum cutoff value to characterize strains and isolates that cluster together. The two-dimensional PCA scatter plot, represented by two axes, the PC1 associated with the *x*-axis and PC2 associated with the *y*-axis. The percent variability represented by PC1 and PC2 is denoted on each axis. The relative positions of the isolates/strains were drawn by the R package ggplot with PC1 representing the x- and PC2 representing the *y*-axis of the scatter plot. These plots allowed the identification of viruses that cluster together in the PCA dendrogram and subsequent categorization of these isolates to groups within the PCA scatter plot. The average VNT for each cluster was superimposed on the PCA scatterplot for further characterization of clusters. Collectively, by superimposing both plots together, the spatial orientation of isolates was assessed by clustering into potential antigenic groups and the VNT average of each group was included. All the analyses were conducted by R (version 3.6.1).

## 3. Results

**Figure 1:**
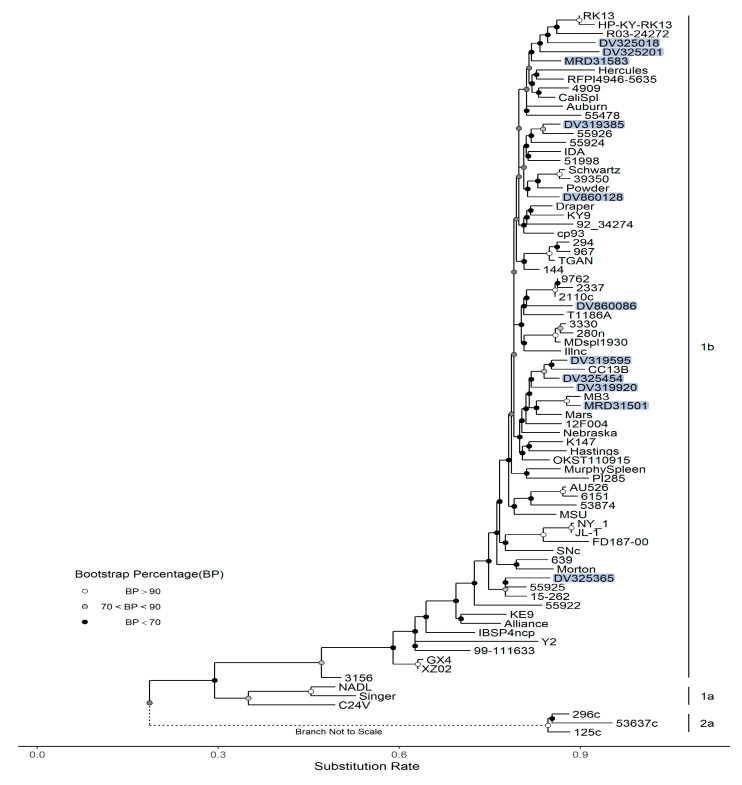
Phylogenetic analysis of the E2 coding sequence of BVDV. Phylogenetic analysis of the E2 sequences of 83 BVDV isolates (3 BVDV-1a, 77 BVDV-1b, and 3 BVDV-2a) to determine amino acid differences within the E2 protein among strains for the major neutralizing protein. Shading represents the 11 BVDV PI strains isolated in this study.

The phylogeny included 83 individual viral strains for representation of genetic diversity, with 77 strains belonging to the BVDV-1b subgenotype and three belonging to the BVDV 1a and three 2a subgenotypes (Figure 1). The 11 BVDV PI strains isolated within the United States and used in this study were dispersed across the phylogenetic tree as depicted by shading in Figure 1. Strain 860,128 was distinct from other BVDV-1b PI strains used in this study, with its closest neighbor being strain Nebraska. Strains 319385 and 325271 were closely related and formed a clade with 55926. Strains 320705 and 325201 formed a clade together, but also formed a larger clade with 2784 and 4090, a strain used in antisera generation. Strains 319393 and 860086 also formed a clade together and a larger clade encompassing Hercules, 9762, 2110c and 2337c, a strain used in antisera generation. Strains 319245, 319595, 325082, 325454, 319920 and 850890 were all close neighbors on the tree, forming a clade with CC13, MB3, and 31501, a strain used for antisera generation. Strains 325365 and 325483 formed a clade together, along with 55925 and 15262. Strains Illnc, 92-34274, 31583, 967, 144, and 3330, all used in antisera production, were genetically distinct and did not form clades with any of the BVDV PI isolated strains. More diverse strains such as GX4, Alliance, Y2 and 3156, not used in antisera production, were very distinct on the tree, and were not closely related to any of the BVDV PI isolated strains.

**Figure 2:**
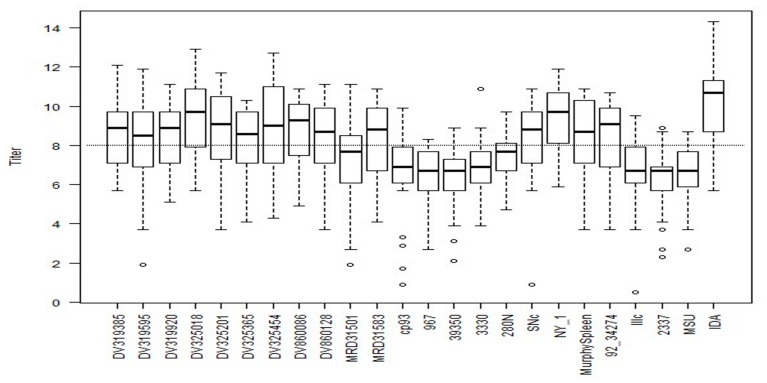
Box plot for neutralizing antibody titers using 24 BVDV-1b isolates against antisera generated against all 24 BVDV-1b isolates.

**Figure 3:**
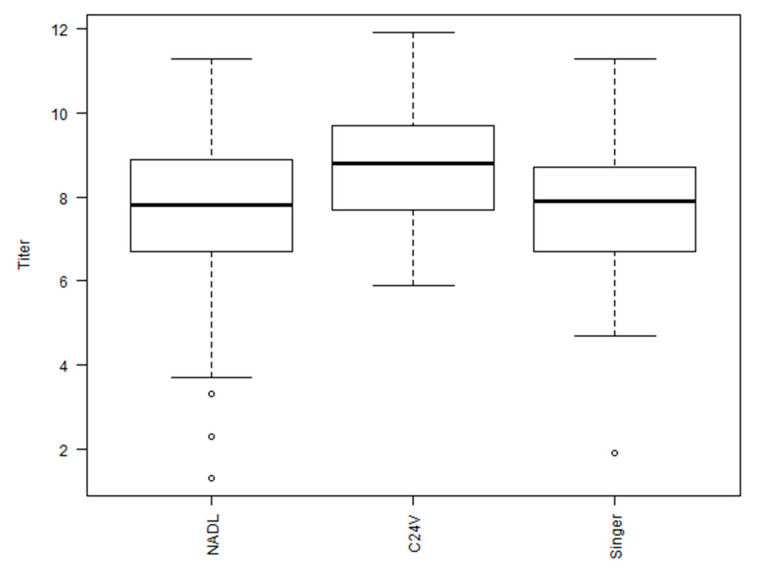
Box plot for neutralizing antibody titers using 24 BVDV 1b isolates against antisera generated against three BVDV 1a vaccine strains (C24V, Singer, and NADL).

**Figure 4:**
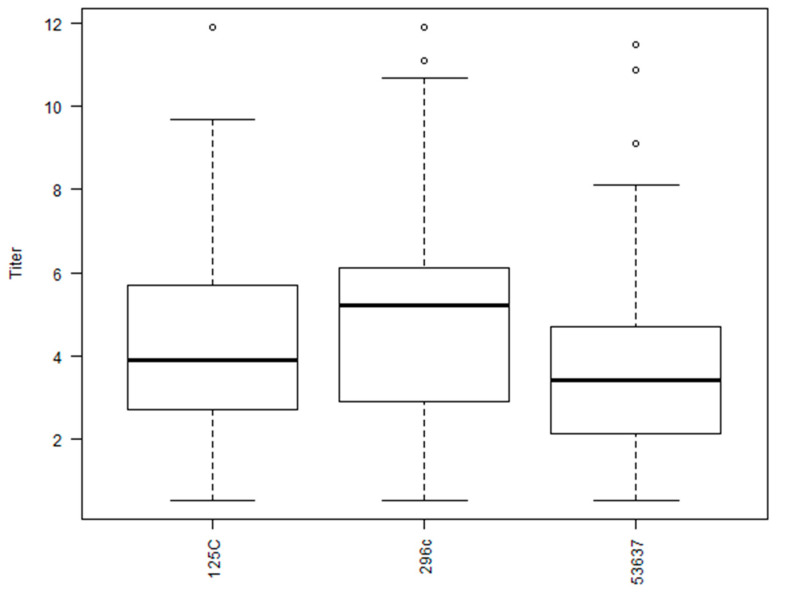
Box plot for neutralizing antibody titers using 24 BVDV 1b isolates against antisera generated against three BVDV 2a vaccine strains (53637c, 125c, and 296c).

The greatest log2 VNT observed among the BVDV-1b antisera against the 30 viruses was IDA (9.9) and the antisera with the lowest VNT was observed with multiple antisera against the 30 viruses, that included 967, 39,350, Illc, 2337, and MSU (6.4, 6.4, 6.8, 6.3, and 6.4, respectively; Figure 2). The average VNT for the twenty-four BVDV-1b antisera against the 30 viruses was 8.0, as denoted by a dashed line, with 9 of the 24 antisera having a VNT less than the average VNT (8.0; Figure 2). The average VNT for the three BVDV-1a antisera against the 30 viruses was 7.9 with C24V having the highest VNT (8.8) and NADL antisera was the lowest (7.3; Figure 3). The average VNT for the three BVDV-2a antisera against the 30 viruses was 4.5 with 296c having the highest VNT (5.1) and 53,637 antisera was the lowest (3.9; Figure 4).

**Figure 5:**
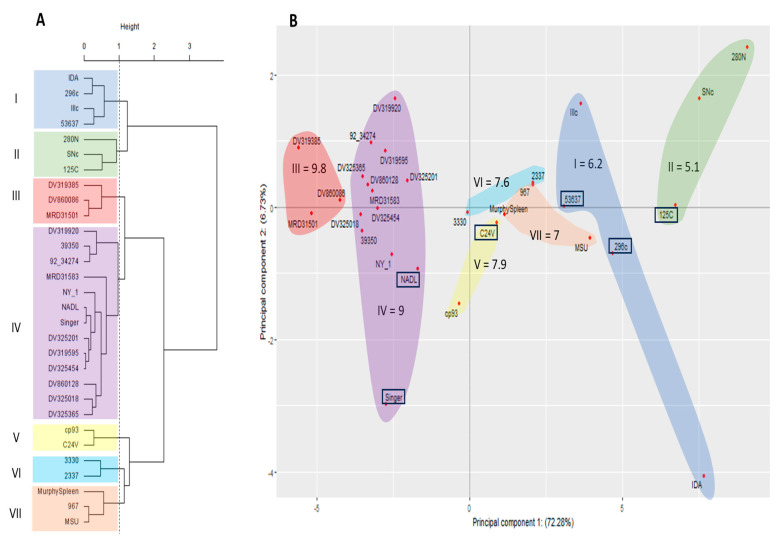
Methods to evaluate similar antigenic clustering using 30 BVDV isolates (3 BVDV-1a, 24 BVDV-1b, and 3 BVDV-2a) and 24 antisera generated against BVDV-1b isolates. (**A**) Cluster analysis dendrogram using Ward’s method combing the variation from both principal component 1 and 2 to cluster strains into like groups. (**B**) Principal component scatter plot displaying independent contribution of the first two principal components accounting for the largest variation in the samples. Vaccine strains denoted by box.

To identify clusters with similar neutralization patterns or divergent viruses, antigenic comparisons using the PCA were evaluated using the antisera from the 24 BVDV-1b viruses against all 30 viruses (Figure 5). Four BVDV-1b viruses (IDA, Illc, 280N, and SNc) were contained in clusters I and II, suggesting these isolates are as antigenically different as the BVDV 2a vaccine viruses when using BVDV-1b antisera. This resulted in a total of seven viruses represented in the antigenically divergent branch as compared to twenty-four viruses represented in the antigenically similar branch. The PCA scatter plot demonstrated the spatial position of the viruses in relation to each other, as well as the position on the PC1 and PC2 axis (Figure 5B). The PC1 (*x*-axis) in the PCA scatter plot was representative of 72.28% of the variability and PC2 (*y*-axis) accounted for 6.73% of the variability (Figure 5B). Interestingly, the BVDV-1a vaccine virus C24V and BVDV-1b viruses MurphySpleen, 967, 2337, and MSU were clustered together in an antigenically similar branch in the PCA dendrogram; however, these viruses are spatially distributed to the right of PC1 axis, indicating they are less similar than viruses distributed to the left of the PC1 axis. Other viruses spatially distributed to the right of PC1 axis, are BVDV-2a vaccine viruses 125c, 296c, and 53637 and BVDV-1b viruses Illc, IDA, SNc, and 280N, although these viruses were included in the antigenically dissimilar branch and clusters. The number of viruses (*n* = 12) spatially distributed to the right of the PC1 axis include more than the number (*n* = 7) represented by the PCA dendrogram. Additionally, clusters (I, II, V, VI, and VII) observed to the right of PC1 are representative of clusters of viruses that have log2 VNT less than 8, which is the average VNT for all strains when using BVDV-1b antisera.

**Figure 6:**
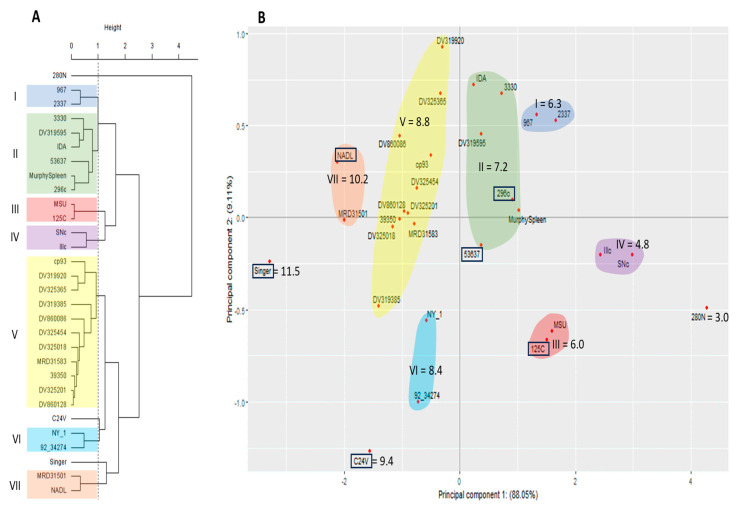
Methods to evaluate similar antigenic clustering using 30 BVDV isolates (3 BVDV-1a, 24 BVDV-1b, and 3 BVDV-2a) and antisera generated against three BVDV 1a vaccine strains (C24V, NADL, and Singer). (**A**) Cluster analysis dendrogram using Ward’s method combing the variation from both principal component 1 and 2 to cluster strains into like groups. (**B**) Principal component scatter plot displaying independent contribution of the first two principal components accounting for the largest variation in the samples. Vaccine strains denoted by box.

Antigenic comparisons using the PCA were also evaluated using the antisera from three BVDV-1a vaccine viruses against all 30 viruses. Using the PCA cluster dendrogram only one BVDV-1b virus (280N) was represented in antigenically divergent branch (Height > 1; Figure 6A), but no BVDV-2 viruses were included in the antigenically divergent branch. All other viruses were contained in clusters within the antigenically similar branch (Heigh < 1; Figure 6A). PC1 (*x*-axis) in the PCA scatter plot was representative of 88.05% of the variability and PC2 (*y*-axis) accounted for 9.11% of the variability (Figure 6B). While the BVDV-2a vaccine viruses and nine BVDV-1b viruses (967, 2337, 3330, DV319595, IDA, MurphySpleen, MSU, SNc, and Illc) were included in the antigenically similar branch in the PCA dendrogram, these viruses are spatially distributed to the right of PC1 axis, indicating they are less similar than viruses distributed to the left of the PC1 axis. Additionally, viruses contained in clusters I, II, III, and IV, observed to the right of PC1 are representative of clusters of viruses that have log2 VNT less than 7.9, which is the average VNT for all strains when using BVDV-1a vaccine antisera.

**Figure 7:**
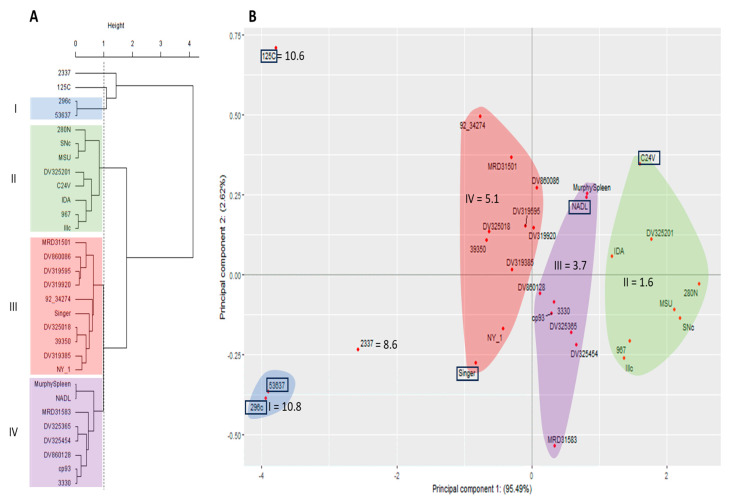
Methods to evaluate similar antigenic clustering using 30 BVDV isolates (3 BVDV-1a, 24 BVDV-1b, and 3 BVDV-2a) and three BVDV 2a antisera generated against vaccine strains (296c 53637c, and 125c). (**A**) Cluster analysis dendrogram using Ward’s method combing the variation from both principal component 1 and 2 to cluster strains into like groups. (**B**) Principal component scatter plot displaying independent contribution of the first two principal components accounting for the largest variation in the samples. Vaccine strains denoted by box. Vaccine strains denoted by box.

Lastly, antigenic comparisons using the PCA were also evaluated using the antisera from three BVDV-2a vaccine viruses against all 30 viruses. The PCA cluster dendrogram, was again represented by two main branches (Figure 7A), with one of the main branches comprised by cluster I that included two BVDV-2a vaccine viruses (296c and 53637) and two other smaller individual branches representing the other BVDV-2a vaccine virus (125c) and BVDV-1b virus (2337). The other main branch that lacked similarity was comprised of three clusters (II, III, and IV). These clusters were comprised of BVDV-1a vaccine viruses and all BVDV-1b viruses excluding 2337 virus. The PCA scatter plot provided further categorization based on the spatial distribution of the viruses and proximity to the PC1 axis (Figure 7). The PC1 (*x*-axis) in the PCA scatter plot was representative of 95.49% of the variability, and PC2 (*y*-axis) accounted for 2.62% of the variability (Figure 7B). While the BVDV-1a vaccine virus Singer and BVDV-1b viruses 2337, MRD31501, DV319595, 92_34274, DV325018, 39350, DV319385, and NY_1 was included in the antigenically similar branch in the PCA dendrogram, these viruses are spatially distributed to the right of PC1 axis, indicating they are less similar than viruses distributed to the left of the PC1 axis. Other viruses spatially distributed to the left of PC1 axis, are the BVDV-2a vaccine viruses 125c, 296c, and 53637 and BVDV-1b virus 2337. The number of viruses (*n* = 12) spatially distributed to the left of the PC1 axis include more than the number (*n* = 4) represented by the PCA dendrogram main branch. Additionally, clusters (II and IV) observed to the right of PC1 are representative of clusters of viruses that have log2 VNT less than 4.5, which is the average VNT for all strains when using BVDV-2a vaccine antisera.

## 4. Discussion

Current licensed commercially available BVDV vaccines in the United States do not contain the BVDV-1b subgenotype [22], leading to questions regarding the effectiveness of available vaccines to prevent infections with BVDV-1b strains [4,13,22]. Although multiple studies have demonstrated effectiveness of commercially available vaccines to confer both a reduction in clinical symptoms and fetal protection [23,24,25,26], suggesting protection is likely due to a variety of factors potentially linked with variation in key antigenic sites within the virus. It is generally considered that the VNT are a result of antibodies against the E2 protein of pestiviruses, which is highly immunogenic [27,28,29]. Interestingly, there does not appear to be a correlation between genetic similarity and antigenic similarity as observed in the current study and previous studies [17,18,19]. In the current study, this is highlighted by the comparisons of E2 genetic phylogeny to the PCA and the lack of similarities among genetic and antigenic clusters.

In this study, multiple PCA using different antisera combinations were evaluated to better understand the impact of antisera generated against vaccine and BVDV-1b viruses. Using VNT, the PCA has been demonstrated to identify antigenic similarities and differences between strains irrespective of phylogeny [17,18,19,30]. Unsurprisingly, data from this study would demonstrate there is variation between vaccine strains and BVDV-1b isolates Although variation was also observed when using the BVDV-1b antisera, also suggesting that BVDV isolates belonging to the same subgenotype (i.e., BVDV-1b) can differ antigenically. Furthermore, eight BVDV-1b viruses were considered antigenically divergent using the PC1 axis in the PCA scatter plot as criteria when using the 1b antisera as compared to nine BVDV-1b viruses when using the same criteria and BVDV-1a vaccine antisera. It should be considered that 24 BVDV-1b antisera compared to three BVDV-1a vaccine antisera against the 30 viruses were included in each respective PCA. It could be hypothesized that the diversity in the BVDV-1b antisera may have contributed to the antigenic diversity that was observed and may not be reflective of the actual antigenic diversity if a more uniform group of BVDV-1b antisera was used. To test this hypothesis, a subsequent analysis using the four highest-titer BVDV-1b antisera 9.9, 9.5, 9.3, and 9.1 corresponding to IDA, DV325018, NY-1 and DV325454, respectively were used for the PCA (Figure S1).

A similar result was observed when using the twenty-four BVDV-1b antisera. Nine BVDV-1b viruses were considered antigenically divergent using the PC1 axis in the PCA scatter plot as criteria. The same BVDV-1b viruses MurphySpleen, 967, 2337, MSU, Illc, IDA, SNc, and 280N were again considered antigenically divergent in addition to 3330. This would highlight that the potential antigenic diversity observed among BVDV-1b viruses is broad and the inclusion of a single BVDV-1b strain in vaccines may not provide the necessary antigenic breath against all BVDV-1b viruses. Data from the current study suggest that current vaccines containing BVDV-1a strains would be antigenically similar to these viruses isolated from PI calves used in this study and the lack of antigenicity may not have been the primary reason associated with the lack of protection. It should be noted that currently licensed vaccines were used in the herds these PI calves were detected, and further demonstrates that administration of a vaccine does not guarantee immunization and immunization does not guarantee prevention of infection. Highlighting that there are many factors that contribute to eliciting a protective response against each respective viruses but understanding the individual contribution of each factor contributing to protection helps identify gaps and potential areas that could be improved. Collectively, data from the current study and previous PCA suggest antigenic diversity expands beyond genetic characterization and beyond one or two viral strains encompassing the breath of antigenicity associated with BVDV. Furthermore, the collective data would suggest that a useful strategy and a greater focus for vaccine strain selection would be to assess antigenic diversity in addition to genetic diversity.

## Figures and Tables

**Table 1:** Viruses used in this study for antisera generation and 30 × 30 analysis with referred strain name and genetic classification.

Category	Virus Name	Subgenotype
BVDV-1a vaccine strains	C24V	BVDV-1a
	NADL	BVDV-1a
	Singer	BVDV-1a
BVDV-2a vaccine strains	125c	BVDV-2a
	296c	BVDV-2a
	53637	BVDV-2a
Isolated from PI calves used for antisera generation and 30 × 30 analysis	DV319385	BVDV-1b
	DV319595	BVDV-1b
	DV319920	BVDV-1b
	DV325018	BVDV-1b
	DV325201	BVDV-1b
	DV325365	BVDV-1b
	DV325454	BVDV-1b
	DV860086	BVDV-1b
	DV860128	BVDV-1b
	MRD31501	BVDV-1b
	MRD31583	BVDV-1b
USDA-ARS-NADC isolates used for antisera generation and 30 × 30 analysis	cp93	BVDV-1b
	967	BVDV-1b
	39350	BVDV-1b
	3330	BVDV-1b
	280N	BVDV-1b
	SNc	BVDV-1b
	NY-1	BVDV-1b
	MurphySpleen	BVDV-1b
	92-34274	BVDV-1b
	Illc	BVDV-1b
	2337	BVDV-1b
	MSU	BVDV-1b
	IDA	BVDV-1b
Viruses used for E2 genetic characterization	50 viral sequences; Table S1.	BVDV-1b

## Data Availability

The data presented in this study are available on request from the corresponding author.

## Supplementary Materials

The following supporting information can be downloaded at: https://www.mdpi.com/article/10.3390/v15102085/s1, Figure S1: Methods to evaluate similar antigenic clustering using 30 BVDV isolates (3 BVDV-1a, 24 BVDV-1b, and 3 BVDV-2a) and the four highest-titer BVDV-1b antisera generated against isolates IDA, DV325018, NY-1 and DV325454. (A) Cluster analysis dendrogram using Ward’s method combing the variation from both principal component 1 and 2 to cluster strains into like groups. (B) Principal component scatter plot displaying independent contribution of the first two principal components accounting for the largest variation in the samples. Table S1: Viruses used in this study for E2 genetic characterization with strain name and genetic classification.
