# Supplementary material for: Multivariate Analysis as a Method to Evaluate Antigenic Relationships between Bovine Viral Diarrhea Virus 1b Isolates and Vaccine Strains
Source: Viruses. 2023 Oct 13;15(10):2085. doi: 10.3390/v15102085 (PMC10612043; doi:10.3390/v15102085)
Supplement: Supplementary file 1 [file viruses-15-02085-s001.zip › viruses-2628942-supplementary.pdf]

Table S1. Viruses used in this study for E2 genetic characterization with strain name and genetic classification

Category	Virus name	Subgenotype
Viruses used for E2 genetic characterization	RK13	BVDV-1b
	HP-KY-RK13	
	R03-24272	
	Hercules	
	RFPI4946-5635	
	4909	
	CaliSpl	
	Auburn	
	55478	
	55926	
	55924	
	51998	
	Schwartz	
	Powder	
	Draper	
	KY9	
	294	
	TGAN	
	144	
	9762	
	2110c	
	T1186A	
	MDspl1930	
	CC13B	
	MB3	
	Mars	
	12F004	
	Nebraska	
	K147	
	Hastings	
	OKST110915	
	PI285	
	AU526	
	6151	
	53874	

	JL-1	
	FD187-00	
	639	
	Morton	
	55925	
	15-262	
	55922	
	KE9	
	Alliance	
	IBSP4ncp	
	Y2	
	99-111633	
	GX4	
	XZ02	
	3156	

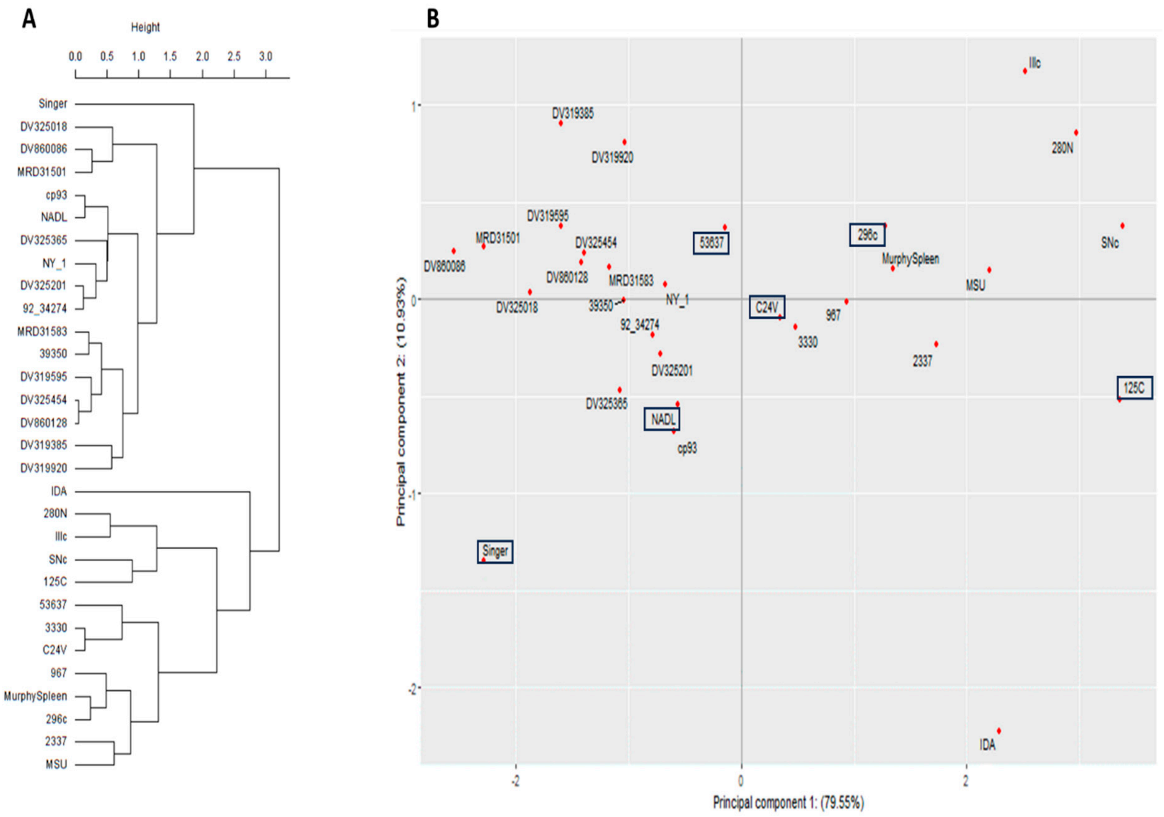

Figure S1. Methods to evaluate similar antigenic clustering using 30 BVDV isolates (3 BVDV-1a, 24 BVDV-1b, and 3 BVDV-2a) and the four highest titer BVDV-1b antisera generated against isolates IDA, DV325018, NY-1 and DV325454. A) Cluster analysis dendrogram using Ward's method combing the variation from both principal component 1 and 2 to cluster strains into like groups. B) Principal component scatter plot displaying independent contribution of the first two principal components accounting for the largest variation in the samples.
